# PP2A: The Wolf in Sheep’s Clothing?

**DOI:** 10.3390/cancers7020648

**Published:** 2015-04-13

**Authors:** Maeve Kiely, Patrick A. Kiely

**Affiliations:** 1 Department of Life Sciences, and Materials and Surface Science Institute, University of Limerick, Limerick 78666, Ireland; E-Mail: Maeve.Kiely@ul.ie; 2 Stokes Institute, University of Limerick 78666, Limerick, Ireland

**Keywords:** phosphatase, PP2A, cellular signaling, tumour suppressor, cancer

## Abstract

Protein Phosphatase 2A (PP2A) is a major serine/threonine phosphatase in cells. It consists of a catalytic subunit (C), a structural subunit (A), and a regulatory/variable B-type subunit. PP2A has a critical role to play in homeostasis where its predominant function is as a phosphatase that regulates the major cell signaling pathways in cells. Changes in the assembly, activity and substrate specificity of the PP2A holoenzyme have a direct role in disease and are a major contributor to the maintenance of the transformed phenotype in cancer. We have learned a lot about how PP2A functions from specific mutations that disrupt the core assembly of PP2A and from viral proteins that target PP2A and inhibit its effect as a phosphatase. This prompted various studies revealing that restoration of PP2A activity benefits some cancer patients. However, our understanding of the mechanism of action of this is limited because of the complex nature of PP2A holoenzyme assembly and because it acts through a wide variety of signaling pathways. Information on PP2A is also conflicting as there are situations whereby inactivation of PP2A induces apoptosis in many cancer cells. In this review we discuss this relationship and we also address many of the pertinent and topical questions that relate to novel therapeutic strategies aimed at altering PP2A activity.

## 1. Introduction

Reversible phosphorylation is a very important mechanism of signal transduction in eukaryotic cells and is mediated by a series of kinases and phosphatases [1,2]. These kinases and phosphatases allow proteins to react rapidly to the external environment by tightly regulating the activity, location and substrate specificity of proteins. Disruption of the regulation of protein phosphorylation can lead to altered cellular behaviour and result in many diseases including cancer [3,4,5,6,7]. Protein Phosphatase 2A (PP2A) is a major serine/threonine phosphatase and is ubiquitously expressed in eukaryotic cells. It is one of the most conserved proteins and together with PP1, is responsible for up to 90% of all serine/threonine activity in a cell [3,8,9]. As a phosphatase, PP2A functions in many of the major cell signaling pathways including those that regulate the cell cycle, cell metabolism, cell migration and cell survival [10,11,12,13].

## 2. PP2A Assembly

PP2A is, in fact, a complex of three specific and individual subunit proteins that function as a holoenzyme. The core dimer consists of a 65 kDa scaffolding subunit (A) and a 36 kDa catalytic subunit (C). In mammals, the A subunit exists in two isoforms (Aα and Aβ) that share almost 87% sequence identity [14,15]. Despite the similarity, approximately 90% of PP2A holoenzymes contain the Aα isoform leaving just 10% of holoenzymes containing the Aβ isoform [16]. This suggests that there is divergent function of the A subunits and is illustrated by the fact that Aβ is unable to substitute for Aα in mice [3]. Aα and Aβ are detected primarily in the cytoplasm but differ in their ability to bind to the B regulatory subunits [17]. Aβ is detected at high levels in embryogenesis but is rare in adult tissues [18] and Aα increases gradually as Aβ decreases after late embryogenesis [19]. Two isoforms of the catalytic subunit also exist (Cα and Cβ). They share 97% sequence identity at the amino acid level. Cα localises primarily to the plasma membrane with Cβ localising primarily to the cytoplasm and nucleus [12,15,20]. Both isoforms have been detected in many rat and porcine tissue types including brain, heart, liver, kidney and ovaries and they are most abundant in the brain and heart [15,21].

**Figure 1:**
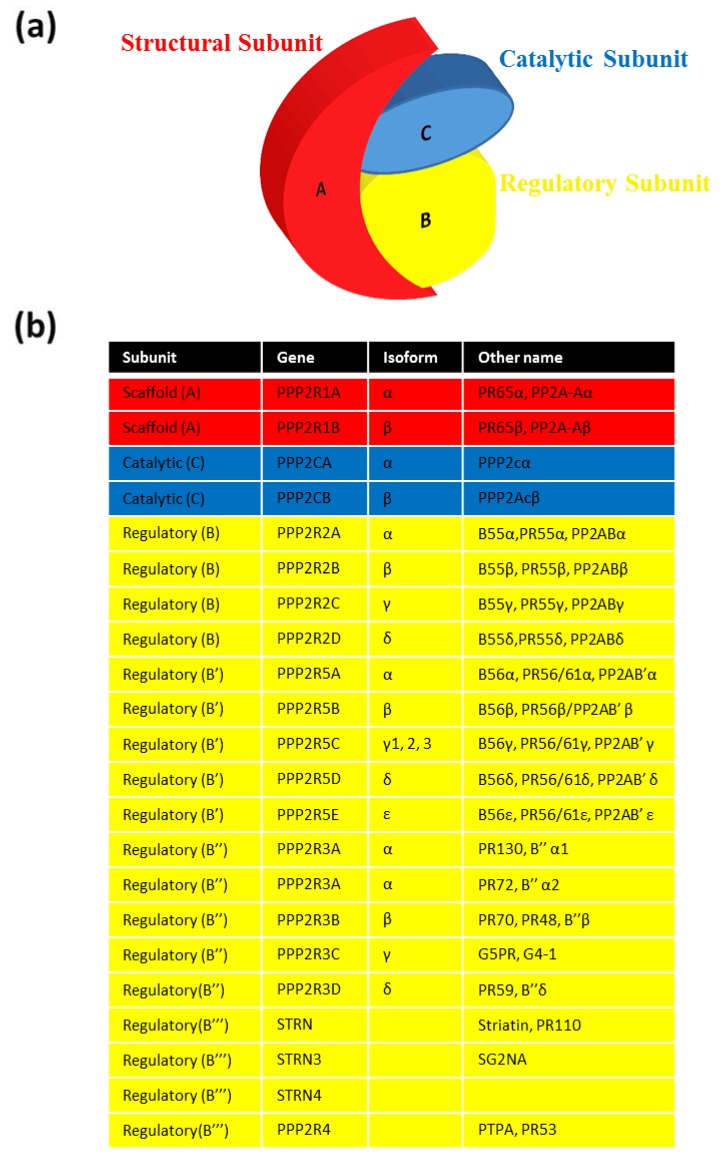
PP2A holoenzyme structure. (**a**) Schematic representation of the Structural (A), Regulatory (B) and Catalytic (C) subunits which form the PP2A holoenzyme. (**b**) Table showing the subunit, gene, isoform and alternative name for each subunit.

Full activity, specific subcellular location and substrate specification is conferred upon PP2A only when the core dimer interacts with the B regulatory subunit to form a heterotrimeric holoenzyme [22] (Figure 1a). In humans, at least 26 different B subtypes have been identified to date [12]. These proteins are alternate transcripts and splice variants encoded by at least 15 different genes [3,12] (Figure 1b). This allows up to 96 possible combinations of the assembled holoenzyme [23]. The expression level of these subunits is highly variable and is dependent on both cell and tissue type [24]. The diversity in regulatory subunit availability is primarily responsible for the widespread substrate specificity and function of the PP2A holoenzyme. As well as this, the PP2A holoenzyme has the ability to interchange B subunits rapidly, allowing PP2A to respond quickly to specific environmental cues [23]. The variable regulatory B subunits are categorized into four recognised subfamilies; B/B55/PR55, B’/B56/PR61, B’’/B72/PR72 and B’’’/PR93(SG2NA)/PR110(Striatin). In contrast to the A and C subunits, the sequence and structural similarity amongst the four regulatory subfamilies is very low and is a major contributor to the diversity of PP2A holoenzymes location and function [3,15,25].

A large body of work has been done to determine specific functions of known PP2A regulatory subunits and is reviewed extensively [3,12,16,26,27]. PP2A-B56 is the best studied regulatory subunit subfamily [3,25]. PP2A-B56α, PP2A-B56β and PP2A-B56ε are expressed in the cytoplasm of cells. The PP2A-B56y1-3 isoforms are expressed in the nucleus while PP2A-B56δ is expressed in both the cytoplasm and nucleus [3]. Substrates of the PP2A holoenzyme containing B56 include the basic proteins, shugoshins (Sgo1) [28,29]. Together, PP2A and Sgo1 function to protect centromeres in mammalian cells from premature chromosome segregation during both mitosis and meiosis. [3,25]. The PP2A-B55 regulatory subunit subfamily have also been well-studied. PP2A-B55α and PP2A-B55δ are, for the most part, expressed ubiquitously. In rat tissues, PP2A-B55β and PP2A-B55y are highly expressed in the brain and are tightly controlled during development [30]. Some of the proteins that have been shown to directly interact with the PP2A-B55 enzyme include Akt, p53, SRC, RAF1, pRB, p107, p130, CDK1 and KSR1 [3,31,32]. In humans, the functions of PP2A-B72 is most widely reported in the regulation of the G1/S part of the cell cycle and the regulation of DNA replication during S phase [3]. Striatin, a member of the B’’’ subfamily is found in high numbers in post synaptic membranes while S/G2 Nuclear Autoantigen (SG2NA), another member of the B’’’ subfamily is targeted to the nucleus and expressed during the S/G2 phases of the cycle [23].

## 3. PP2A: Disrupt the Core, Decrease the Function

Mutations in PP2A subunits have been found in a variety of human cancers and include deletions, point mutations and mutations that generate alternate transcripts [13,33]. Some of these mutations prevent the A subunit from binding to the B and C subunits. This can cause disruption of the core complex which has implications for PP2A activity [15,34,35,36]. The gene encoding the Aα subunit, PPP2R1A, is mutated in over 40% of high grade endometrial tumours [37]. PPP2R1A has also been shown to be mutated in 9.1% of Type I ovarian tumours and 6.8% of Type I uterine carcinomas [38]. As well as this, specific mutations of PPP2R1A have been identified in lung carcinoma, breast carcinoma and melanoma. These mutations result in a decreased binding to other PP2A subunits [35,39]. In mice expressing two Aα mutations (E64D and E64G), the presence of either mutation resulted in defective binding of the Aα subunit to B subunits [40]. Mice expressing the E64D mutation demonstrated increased incidence of p53 dependant lung cancer [40]. This study highlights the role of PP2A as a tumour suppressor and suggests that disruption of the PP2A holoenzyme may contribute to the development of carcinogenesis.

PPP2R1B, the gene encoding the Aβ subunit of PP2A is located at 11q23 which is a chromosomal region that is frequently deleted in cancer cells [41]. PPP2R1B is mutated in 15% of both primary lung tumours and colorectal carcinomas [42]. One PPP2R1B mutation seen in 6% of lung cancer cell lines, is defective in binding to the PP2A C subunit [42]. Five missense mutations of PPP2R1B have been identified in colorectal tumours [43]. Other PPP2R1B mutations have been detected in human hepatocellular carcinomas [44] and are also found in melanoma and cancers of the breast and lung [39].

PP2A B subunit mutations are less frequent, but some deletion mutations have been identified in breast, prostate and ovarian cancers [33,45,46,47]. Reduced expression of PP2A-B55α has been reported in leukaemia [48]. PP2A-B55β is epigenetically inactivated by DNA hypermethylation in colorectal cancer which has consequences for myc signaling [49]. PP2A-B55β has also been shown to be mutated by hypermethylation in breast cancer [50].

## 4. Displacing the Regulatory Subunit: What We Have Learned from Viral Targeting of PP2A and from PP2A Inhibition?

There is very strong evidence to suggest that specific PP2A holoenzymes are genuine tumour suppressors. This tumour suppressor function for PP2A was realised when it was shown that okadaic acid, a well-known tumour promoter, is a selective (albeit non-specific) inhibitor of PP2A [51]. PP2A has a regulatory function in many signaling pathways that control cell growth, cell migration and apoptosis. This makes it an obvious target for oncogenes targeting deregulation of the cell cycle. In transformed cells, several mechanisms have evolved that inhibit PP2A and target carcinogenesis [52]. For example, some DNA tumour viruses produce viral proteins that displace B subunits from PP2A and can function as pseudo B subunits themselves [3,53]. This results in altered activity and location of PP2A, leading to dysregulation of the signaling pathways where PP2A normally functions [3]. Retroviruses such as HIV1 VPR also interfere with PP2A to induce cell cycle arrest [53,54].

**Figure 2:**
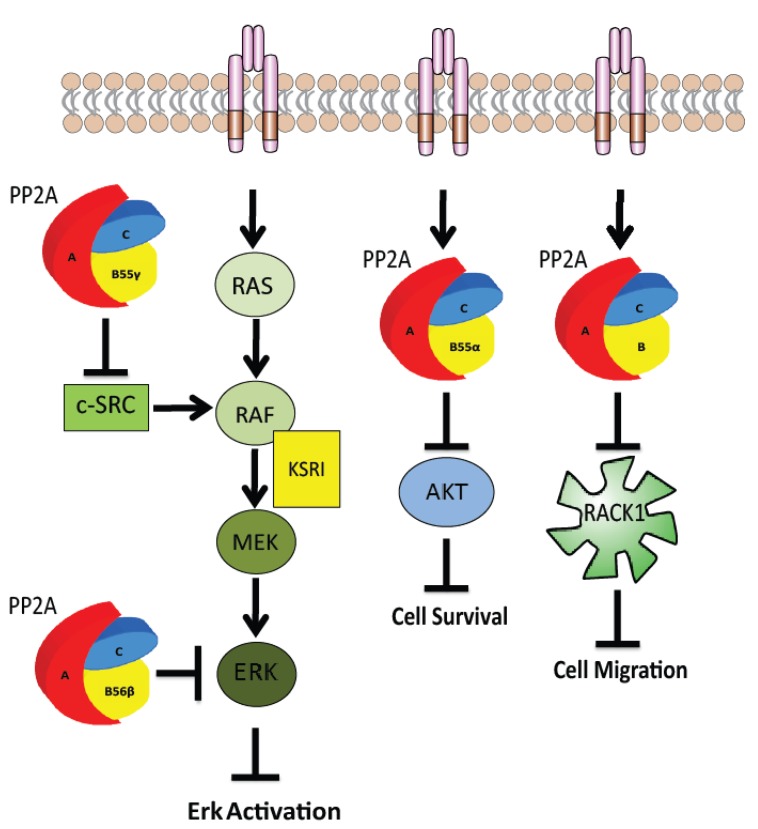
PP2A as a tumour suppressor in signaling pathways. The PP2A holoenzyme containing B55γ, negatively regulates the MAP kinase pathway through inhibition of c-SRC [3,69,70]. PP2A holoenzymes containing B56β and B56γ directly dephosphorylate ERK to negatively regulate the MAP kinase pathway [59]. PP2A negatively regulates Akt activity through dephosphorylation of Akt at Thr 308 to regulate cell survival [71]. As well as functioning as a phosphatase, PP2A also controls cell migration by acting as a regulator of growth factor and adhesion receptor assembly through its interaction with RACK1 during cell migration [72].

Polyoma small t (pyst), polyoma middle T (pyMT) and simian virus small t (ST) are viral proteins that can form complexes with the PP2A core dimer [55,56,57,58]. When pyMT displaces the B subunit of the PP2A holoenzyme, the MAP kinase pathway is activated and interaction of pyMT with PP2A is required for pyMT induced transformation [57]. A number of PP2A B subunits inhibit and activate proteins in the MAP kinase signaling pathway including RAF and ERK [3]. PP2A-B56y^1^ dephosphorylates ERK which inhibits ERK activity [59]. PP2A-B55 has been shown in a variety of capacities within this pathway. PP2A-B55α or PP2A-B56 positively regulate RAF1 MAP kinase signaling by dephosphorylating an inhibition site on RAF1. PP2A-B55α dephosphorylates key 14-3-3 binding sites on Kinase Suppressor of RAS (KSR1) which also positively regulates the MAP kinase pathway [32]. These specific holoenzymes can also negatively regulate MAP kinase signaling by dephosphorylating and deactivating ERK further downstream of RAF [60] (see Figure 2). The ability of pyMT to specifically activate the MAP kinase pathway suggests that deregulation of PP2A assembly and disruption of PP2A-B55α or PP2A-B56 binding to the holoenzyme promotes deregulated growth control [61].

Small t antigen (ST) is a simian virus 40 ER viral onco-protein which when co expressed with large T antigen, the telomerase catalytic subunit and the H-RAS oncogene, is able to transform human cells. ST requires interaction with PP2A in order for this transformation to occur [62,63,64]. ST binds to the Aα subunit to regulate the Akt pathway [3]. ST also displaces PP2A-B55α, PP2A-B56y and PP2A-B56y3 regulatory subunits and when bound to the core subunit, ST will suppress PP2A activity [65]. Displacement of a subset of these PP2A complexes (PP2A-B56γ and PP2A-B56γ3) increases cell proliferation and confers an ability to grow in an anchorage independent manner leading to tumour development in animal hosts [65]. Induction of increased cell proliferation in cells where PP2A has been displaced by ST has previously been shown to be mediated by stimulation of the MAP kinase pathway [66,67]. ST, when interacting with PP2A, directly regulates the dephosphorylation of AKT. In cells where growth factors are absent, ST interaction with PP2A enhances the anti-apoptotic activity of Akt. Conversely, in the presence of growth factors, ST interaction with PP2A enhances the pro-apoptotic activity of Akt. [68].

Okadaic acid is a well-known PP2A inhibitor and a potent carcinogen [51,73,74]. There are several other mechanisms of PP2A inhibition that contribute to carcinogenesis. Cancerous Inhibitor of PP2A (CIP2A) is a PP2A inhibitor which when over expressed, is associated with poor outcome [75,76]. Specifically, CIP2A inhibits the activity of PP2A which promotes stabilisation of the known oncogenic transcription factor c-Myc through phosphorylation on Ser62. CIP2A also promotes anchorage independent growth and tumour formation [76]. Transcription of CIP2A is decreased when the checkpoint kinase and DNA damage response protein, CHK1 is inhibited. This induces PP2A activity allowing dephosphorylation of c-MYC on Ser62 which impairs cancer cell survival [77]. Overexpression of SET, which in many cases is known to be a potent PP2A inhibitor is associated with poor prognosis in Acute Myeloid Leukaemia (AML) [78]. Other PP2A inhibitors known to progress carcinogenesis include cytostatin [79] and rubratoxin A which inhibits PP2A and suppresses metastasis [80].

## 5. PP2A: A Key Regulator of Growth Factor Signaling

PP2A has also been shown to play a considerable role in the regulation of growth factor signaling [67,72,81,82,83]. Stimulation of cells with EGF, insulin and IGF-1 inhibits PP2A activity and promotes sustained activation of the MAP kinase pathway [67]. The growth factor stimulation causes disassociation of PP2A from the adaptor protein Shc. This leads to increased and sustained Shc phosphorylation and subsequent activation of the MAP kinase pathway to promote cell proliferation [67]. In gastric cancer cells, the EGFR positively regulates the expression of the PP2A inhibitor CIP2A [84] to drive metastasis.

Downstream of the Insulin receptor, PP2A directly dephosphorylates Akt on Ser473 and Thr308 to negatively regulate metabolic signaling [81,83]. The PP2A-B56 regulatory subunit has been implicated in the negative regulation of insulin signaling through Akt dephosphorylation at Thr308 [85]. However, the PP2A holoenzyme containing PP2A-B55α has also been identified as a regulator of Akt phosphorylation at Thr308 acting to negatively regulate Akt mediated cell proliferation and survival [71]. In breast cancer cells, PP2A associates with the scaffolding protein RACK1 in an IGF-1 dependant manner and regulates cell migration and proliferation by controlling the formation of a complex between the IGF-IR, RACK1 and β1 integrin [72,82]. In this context, it is not PP2A activity that is the key regulator; rather, it is the ability of PP2A to compete with β1 Integrin for binding to Tyr302 of RACK1.

## 6. Therapeutic Strategies that Restore PP2A Activity

Even subtle changes in expression or activity of phosphatases may lead to a diseased state. In cancer, phosphatases present as a major focus of therapeutic targets as they are deregulated in almost all cancer types [7], and strategies that restore activity of PP2A merit serious consideration as novel treatments for cancer [86]. There are a number of compounds available that, although not exclusively targeting PP2A alone, have been shown to reverse the action of PP2A inhibition, to restore PP2A activity levels. Forskolin, most well known as an activator of adenylyl cyclase activity, has also been used to reduce growth of AML cells and induce apoptosis through caspase dependant mechanisms resulting from activation of PP2A [87]. Ceramide is a bioactive lipid which activates PP2A through targeting of the interaction between PP2A and SET and has been shown to induce apoptosis in cancer cells [88].

FTY720 is a sphingosine analog drug and a potent immunosuppressant that has been approved by the FDA as a treatment for multiple sclerosis (MS) after a series of successful clinical trials [89,90,91,92,93]. FTY720 is an activator of PP2A [94] and this mechanism of action has shown promise as an anti-cancer therapy in many pre-clinical studies. These studies have investigated the use of FTY720 as a potential therapy for a number of cancer cell types including but not limited to, neuroblastoma, bladder, renal, colorectal, breast, ovarian and lung cancers [58,95,96,97,98,99,100] and an *in vivo* study of renal cancer using mouse models [97]. In colorectal cancer, PP2A inactivation is a common occurrence and is accompanied by up-regulation of a number of well-known PP2A inhibitors including CIP2A [58]. Treatment of colorectal cancer cell lines with FTY720 shows reduced proliferation and an increase in the pro-apoptotic factors caspase-3 and caspase-7 and appears to be accompanied by an increase in PP2A expression [58]. As well as this, treatment of colorectal cancer cells with FTY720 enhances the effect of a number of well-established chemotherapeutic agents such as 5-fluorouracil and oxalipatin [58].

One pre-clinical study showed that oestrogen receptor negative (ER−) breast cancer cell lines were more sensitive to FTY720 than cells that express the oestrogen receptor (ER+) [101]. This is attributed to ER− breast cancer cell lines having suppressed levels of PP2A activity in comparison to ER+ breast cancer cell lines thus having a higher sensitivity to a PP2A activator such as FTY720. This predictive study suggests a potential assessment and treatment strategy for patients, to identify and target a subset of patients with reduced PP2A with a PP2A activator such as FTY720.

Many pre-clinical studies have focused on the effect of FTY720 on leukaemia cell lines. A number of myeloid leukaemia (AML) cell lines are sensitive to FTY720, particularly those with a specific D816V mutation in the tyrosine kinase domain of C-KIT [86,102]. Cell lines with this mutation show inhibition of PP2A activity with decreased expression of the A subunits of PP2A, PP2A-B55α and PP2A-B56α, γ and δ [102]. The toxic effect of FTY720 in these cells is mediated by reactivation of PP2A. This reactivation occurs via downregulation of the PP2A inhibitor SET, upregulation of the PP2A-A subunit and PP2A-B55α, and dephosphorylation of the PP2A C subunit [86]. Restoration of PP2A activity then promotes the induction of apoptosis and inhibition of proliferation [86,102]. Over expression of PP2A Aα in cells harbouring the D816V mutation also induces apoptosis and inhibits proliferation [102].

## 7. The Anti-Apoptotic Role of PP2A

The predominant perception of PP2A functioning solely as a tumour suppressor and a regulator of pathways that promote apoptosis is being challenged. A growing body of evidence suggests an anti-apoptotic role for PP2A. This was first noted in *Drosophila* [103] and in mammalian cell models where inactivation of PP2A induces apoptosis in a number of cancer cell types including cancers of the pancreas, testes, liver and in leukaemic cells [104,105,106,107,108]. PP2A plays a dual regulatory role in apoptosis, facilitating both pro and anti-apoptotic signaling depending on the holoenzyme assembled and pathways targeted [109,110,111,112]. Particularly well studied is the relationship between Bcl-2 and PP2A. PP2A dephosphorylates Bcl-2, however, depending on the cellular location, this can manifest as either a pro or anti-apoptotic signal [112,113,114]. For example, PP2A-B56α promotes apoptosis through dephosphorylation of Bcl-2 on Ser70 in the mitochondria [112,113]. However, PP2A inhibits apoptosis by dephosphorylating Bcl-2 on Ser87 in tumour cell lines and this inhibition is not seen in normal human blood cells [114]. The specific PP2A regulatory subunits involved in this process are yet to be identified. This presents a good example of the “molecular tightrope” that PP2A walks, and highlights the importance of holoenzyme assembly and the central role being played by the regulatory subunit which regulates the sub-cellular location of PP2A.

p53 is a major and extensively studied tumour suppressor protein that is mutated or deleted in many cancers [115]. Under stress conditions such as DNA damage, p53 is stabilised and accumulated by phosphorylation events conferred by a number of stress activated kinases that induce cell cycle arrest or apoptosis [116]. Ser15 has been identified as an important phosphorylation site in the regulation of p53. A number of phosphatases are known to play a regulatory role by targeting p53 phosphorylation on Ser15. For example, PP1 dephosphorylates p53 at this site as a negative regulator to promote cell survival [117] and Wip1 is involved in cell cycle arrest in tumour cells [118]. PP2A is also a regulator of p53 and dephosphorylates p53 on Ser15. In glioblastoma models, pharmacological inhibition of PP2A decreases p53 abundance and activates AKT-1 and Plk-1 [107]. This inhibition of PP2A blocks the cell cycle arrest and can enhance the efficacy of chemotherapy drugs that work by damaging DNA or disrupting components of cell replication. Conversely, in cervical cancer cells, inhibition of PP2A stabilizes and activates overexpressed p53 [116], resulting in cell cycle arrest and the promotion of apoptosis through up-regulation of Bax and p21. In this case, inhibition of PP2A maintains p53 phosphorylation and therefore promotes its “activity” as a tumour suppressor suggesting an anti-apoptotic role for PP2A [116]. In osteosarcoma cells, PP2A-B56γ3 interacts with p53 through an Ataxia Telangectasia Mutated (ATM) dependant mechanism. Phosphorylation of p53 by ATM at Ser15 promotes PP2A mediated dephosphorylation of p53 on Thr55 to inhibit cell proliferation. This, conversely, is strong evidence in support of a tumour suppressor role for the PP2A holoenzyme containing B56γ [119,120]. Mutations that disrupt the binding of B56y to the A and C subunits in their own right have been shown to disrupt the p53 dependent tumour suppressor functions [121].

## 8. Are There Benefits to Inhibiting PP2A?

Overexpression of the catalytic subunit of PP2A in hepatocellular cancer models and virus infected cells disrupts p53 phosphorylation and inhibits p53 mediated apoptosis [106]. Mouse models that have the catalytic subunit of PP2A overexpressed, have larger and a greater number of hepatocellular tumours suggesting that PP2A has a role to play in tumour progression [106,122]. Studies like these suggest that PP2A should be considered as a therapeutic target in some cancer types.

Lenalidomide is a therapeutic agent, approved by the FDA in 2002 for the treatment of a specific subset of myelodysplastic syndrome (MDS) patients who have an isolated deletion of chromosome 5q (reviewed extensively in [123]). The mechanism of action of lenalidomide is mediated through inhibition of the PP2A Cα subunit resulting in cell cycle arrest and apoptosis in MDS cells with the 5q deletion [124]. Clinical trials have shown treatment with lenolidomide to confer a significant increase in median overall survival [125]. Development of lenolidomide resistance in patients has been attributed to over-expression of the PP2A Cα subunit [123].

Cantharidin is a toxin isolated from *Mylabris phalerata or Mylabris cichorii*. It is a known anti-cancer agent [104] and a potent but non-specific PP2A inhibitor [126]. Its anti-cancer effect, as seen in pancreatic cell models, is mediated through induction of pro apoptotic proteins including caspase-8 and caspase-9 as well as tumour necrosis factor-alpha leading to a subsequent, dose dependant increase in apoptosis. This is accompanied by a simultaneous decrease in the expression of the anti- apoptotic factor Bcl-2 [104]. Inhibition of malignant testicular germ cell tumours using okadaic acid and cantharidin induces an anti-apoptotic effect through phosphorylation and subsequent activation of both MEK and Erk which, in turn, activate one of the most prominent inducers of apoptosis, caspase-3 [105]. In leukaemia cells, PP2A inhibition again leads to activation of caspase-3 as well as activation of caspases-8 and caspase-9 leading to caspase dependant apoptosis, DNA fragmentation and mitochondrial permeabilization [108,127]. However, cantharidin has very toxic side effects at concentrations > 10 µmoL/L and has been associated with severe poisoning and death. This suggests that although it effectively induces apoptosis at lower concentrations (2–5 µM), the associated side effects will more than likely prevent it from ever becoming a mainstream treatment for cancer [128]. A less toxic demethylated analog of cantharidin, norcatharidin, shows a promising ability to induce apoptosis in a number of cancer cell types including melanoma, breast, oral and gallbladder cancers with some effectiveness [129,130,131,132]. Modified components of cantharidin (such as demethylcantharidin) have shown tumour growth suppression (through PP2A inhibition) in both HCC cell lines and xenograft models in nude mice [133]. In cisplatin resistant mouse models, the modified compounds were tested and were found to demonstrate similar levels of tumour growth suppression compared to cisplatin sensitive mouse models. The combination of minimal toxicity and promising potency of these PP2A inhibitors suggest strong potential for these platinum based drugs to be used in the treatment of HCC [134].

Work is continuing in order to develop small molecules that will inhibit PP2A with reduced side effects. LB-102 inhibits PP2A to increase the efficacy of some well-known chemotherapy drugs including doxorubicin in xenograft animal models of glioblastoma. In this situation, inhibition of PP2A blocks the DNA damage defense mechanisms in the cell that is being targeted by the chemotherapeutic agent [107]. LB-100 is currently the subject of a phase 1 clinical trial examining the safety and efficacy of the small molecule in treatment of patients with advanced solid tumours as a result of PP2A inhibition (NCT01837667) [135]. LB-100 has already shown successful anti-proliferative effects in the treatment of both *in vitro* and *in vivo* models of glioblastoma [136]. Inactivation of PP2A, in particular the holoenzymes involving regulatory subunits B56γ and B56δ, induces phosphorylation of the apoptosis inducing protein Apoptin. Apoptin is a protein derived from an avian virus which, when phosphorylated, can induce apoptosis specifically in transformed human cells and not in normal cells [137]. Apoptin has been shown to work in a broad spectrum of transformed cells and is considered to be a potentially safe and effective anti-cancer treatment.

**Figure 3:**
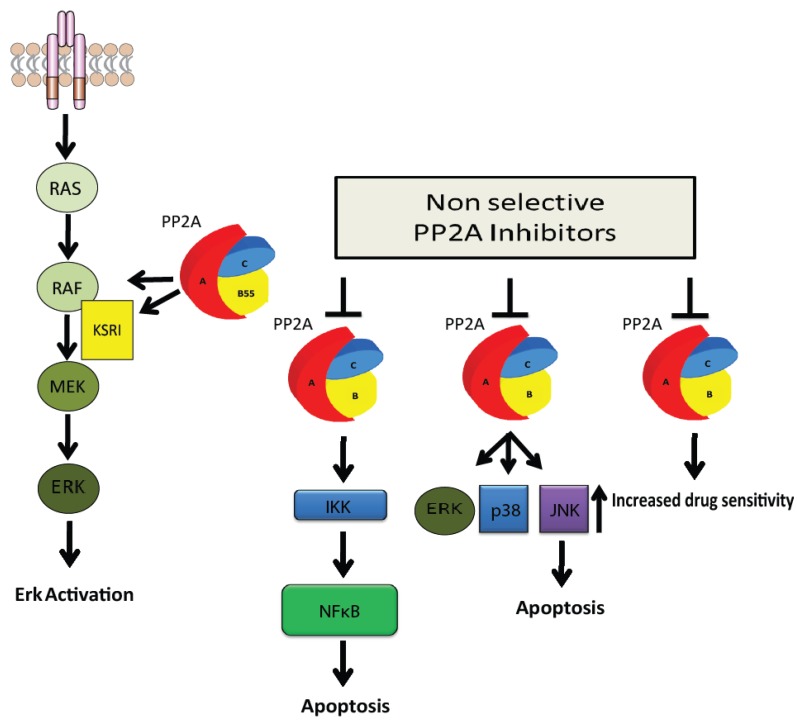
PP2A as a promoter of carcinogenesis. The PP2A holoenzymes containing B55α and B55δ play a positive regulatory role in the MAP kinase pathway through direct dephosphorylation of RAF1 on Ser259. The PP2A holoenzyme containing B55α also dephophorylates KSR1 on Ser392 and RAF on Ser295 to activate ERK [31,32]. Inhibition of PP2A by Cantharidin promotes apoptosis in cancer cells by mediating the prolonged phosphorylation of I κ B kinase α (IKK) and activation of NFκB [145]. Cantharidin also induces apoptosis by blocking PP2A mediated activation of the MAP kinase pathway [81,145]. Inhibition of PP2A using okadaic acid increases the sensitivity of breast cancer cells to the drug lapatinib which has an anti-proliferative effect on cells [138].

Increased PP2A activity has recently been shown to contribute to the mechanism of drug resistance in the HER2 positive subtype of breast cancer [138]. Lapatinib is a drug that is approved to treat patients with HER2 positive, metastatic, trastuzumab refractory breast cancer when used in combination with capecitabine as it has been shown to prolong median survival time [139]. In one study, increased PP2A activity has been identified as a contributory factor in the development of resistance to lapatinib [138]. PP2A activity was found elevated in two lapatinib resistant breast cancer cell lines. Both cell lines, as expected, demonstrated increased sensitivity to OA and OA induced inhibition of PP2A in these cells resulted in increased sensitivity to lapatinib (Figure 3). It was also demonstrated in this study that treatment of a lapatinib sensitive breast cancer cell line with the PP2A activator FTY720, decreased its sensitivity to the drug. Many other mechanisms of laptatinib resistance have been investigated [134,140,141,142,143,144], however the authors of this study suggest that PP2A has potential as a novel biomarker of lapatinib resistance and PP2A inhibition in combination with HER2 inhibition merits further investigation as a potential therapy for this type of breast cancer [138].

## 9. Summary

PP2A is an indispensable enzyme in cells which transverses cellular signaling pathways to regulate a diverse array of proteins. In this review, our objective was to highlight that although best known as a tumour suppressor, PP2A also has a dark side that is slowly emerging. Accumulating evidence points to inactivation of PP2A being an appropriate course of treatment in particular sets of cancer and PP2A has emerged as an attractive therapeutic target in malignancy.

## References

[1] Hunter T. (1995). Protein kinases and phosphatases: The yin and yang of protein phosphorylation and signaling. Cell.

[2] Pawson T., Scott J.D. (1997). Signaling through scaffold, anchoring, and adaptor proteins. Science.

[3] Eichhorn P.J.A., Creyghton M.P., Bernards R. (2009). Protein phosphatase 2A regulatory subunits and cancer. Biochim. Biophys. Acta.

[4] Tonks N.K. (2006). Protein tyrosine phosphatases: From genes, to function, to disease. Nat. Rev. Mol. Cell Biol..

[5] Östman A., Hellberg C., Böhmer F.D. (2006). Protein-tyrosine phosphatases and cancer. Nat. Rev. Cancer.

[6] Gallego M., Virshup D.M. (2005). Protein serine/threonine phosphatases: Life, death, and sleeping. Curr. Opin. Cell Biol..

[7] Stebbing J., Lit L.C., Zhang H., Darrington R.S., Melaiu O., Rudraraju B., Giamas G. (2013). The regulatory roles of phosphatases in cancer. Oncogene.

[8] Arroyo J.D., Hahn W.C. (2005). Involvement of PP2A in viral and cellular transformation. Oncogene.

[9] Westermarck J., Hahn W.C. (2008). Multiple pathways regulated by the tumor suppressor PP2A in transformation. Trends Mol. Med..

[10] Janssens V., Goris J. (2001). Protein phosphatase 2A: A highly regulated family of serine/threonine phosphatases implicated in cell growth and signalling. Biochem. J..

[11] Schönthal A.H. (2001). Role of serine/threonine protein phosphatase 2A in cancer. Cancer Lett..

[12] Seshacharyulu P., Pandey P., Datta K., Batra S.K. (2013). Phosphatase: PP2A structural importance, regulation and its aberrant expression in cancer. Cancer Lett..

[13] Perrotti D., Neviani P. (2013). Protein phosphatase 2A: A target for anticancer therapy. Lancet Oncol..

[14] Hemmings B.A., Adams-Pearson C., Maurer F., Müller P., Goris J., Merlevede W., Hofsteenge J., Stone S.R. (1990). Alpha- and beta- forms of the 65-kDa subunit of protein phosphatase 2A have a similar 39 amino acid repeating structure. Biochemistry.

[15] Chen W., Wang Z., Jiang C., Ding Y. (2013). PP2A-Mediated Anticancer Therapy. Gastroenterol. Res. Pract..

[16] Yang J., Phiel C. (2010). Functions of B56-containing PP2As in major developmental and cancer signaling pathways. Life Sci..

[17] Zhou J., Pham H.T., Ruediger R., Walter G. (2003). Characterization of the Aalpha and Abeta subunit isoforms of protein phosphatase 2A: Differences in expression, subunit interaction, and evolution. Biochem. J..

[18] Bosch M., Cayla X., van Hoof C., Hemmings B.A., Ozon R., Merlevede W., Goris J. (1995). The PR55 and PR65 subunits of protein phosphatase 2A from *Xenopus laevis*. Eur. J. Biochem..

[19] Hendrix P., Turowski P., Mayer-Jaekel R.E., Goris J., Hofsteenge J., Merlevede W., Hemmings B.A. (1993). Analysis of subunit isoforms in protein phosphatase 2A holoenzymes from rabbit and Xenopus. J. Biol. Chem..

[20] Stone S.R., Hofsteenge J., Hemmings B.A. (1987). Molecular cloning of cDNAs encoding two isoforms of the catalytic subunit of protein phosphatase 2A. Biochemistry.

[21] Khew-Goodall Y., Mayer R.E., Maurer F., Stone S.R., Hemmings B.A. (1991). Structure and transcriptional regulation of protein phosphatase 2A catalytic subunit genes. Biochemistry.

[22] Xu Y., Xing Y., Chen Y., Chao Y., Lin Z., Fan E., Yu J.W., Strack S., Jeffrey P.D., Shi Y. (2006). Structure of the protein phosphatase 2A holoenzyme. Cell.

[23] Janssens V., Longin S., Goris J. (2008). PP2A holoenzyme assembly: *In cauda venenum* (the sting is in the tail). Trends Biochem. Sci..

[24] Xu Y., Chen Y., Zhang P., Jeffrey P.D., Shi Y. (2008). Structure of a protein phosphatase 2A holoenzyme: Insights into B55-mediated Tau dephosphorylation. Mol. Cell.

[25] Sents W., Ivanova E., Lambrecht C., Haesen D., Janssens V. (2013). The biogenesis of active protein phosphatase 2A holoenzymes: A tightly regulated process creating phosphatase specificity. FEBS J..

[26] Janssens V., Goris J., van Hoof C. (2005). PP2A: The expected tumor suppressor. Curr. Opin. Genet. Dev..

[27] Kurimchak A., Graña X. (2012). PP2A holoenzymes negatively and positively regulate cell cycle progression by dephosphorylating pocket proteins and multiple CDK substrates. Gene.

[28] Kitajima T.S., Sakuno T., Ishiguro K., Lemura S., Natsume T., Kawashima S.A., Watanabe Y. (2006). Shugoshin collaborates with protein phosphatase 2A to protect cohesin. Nature.

[29] Tanno Y., Kitajima T.S., Honda T., Ando Y., Ishiguro K., Watanabe Y. (2010). Phosphorylation of mammalian Sgo2 by Aurora B recruits PP2A and MCAK to centromeres. Genes Dev..

[30] Strack S., Chang D., Zaucha J.A., Colbran R.J., Wadzinski B.E. (1999). Cloning and characterization of Bδ, a novel regulatory subunit of protein phosphatase 2A. FEBS Lett..

[31] Adams D.G., Coffee R.L., Zhang H., Pelech S., Strack S., Wadzinski B.E. (2005). Positive regulation of Raf1-MEK1/2-ERK1/2 signaling by protein serine/threonine phosphatase 2A holoenzymes. J. Biol. Chem..

[32] Ory S., Zhou M., Conrads T.P., Veenstra T.D., Morrison D.K. (2003). Protein phosphatase 2A positively regulates Ras signaling by dephosphorylating KSR1 and Raf-1 on critical 14-3-3 binding sites. Curr. Biol..

[33] Kurimchak A., Graña X. (2012). PP2A Counterbalances Phosphorylation of pRB and Mitotic Proteins by Multiple CDKs Potential Implications for PP2A Disruption in Cancer. Genes Cancer.

[34] Ruediger R., Pham H.T., Walter G. (2001). Alterations in protein phosphatase 2A subunit interaction in human carcinomas of the lung and colon with mutations in the Aβ subunit gene. Oncogene.

[35] Ruediger R., Pham H.T., Walter G. (2001). Disruption of protein phosphatase 2A subunit interaction in human cancers with mutations in the Aα subunit gene. Oncogene.

[36] Sablina A.A., Chen W., Arroyo J.D., Corral L., Hector M., Bulmer S.E., DeCaprio J.A., Hahn W.C. (2007). The tumor suppressor PP2A Aβ regulates the RalA GTPase. Cell.

[37] McConechy M.K., Anglesio M.S., Kalloger S.E., Yang W., Senz J., Chow C., Heravi-Moussavi A., Morin G.B., Mes-Masson A.M., Australian Ovarian Cancer Study Group (2011). Subtype-specific mutation of PPP2R1A in endometrial and ovarian carcinomas. J. Pathol..

[38] Shih I.-M., Panuganti P.K., Kuo K.T., Mao T.L., Kuhn E., Jones S., Velculescu V.E., Kurman R.J., Wang T.L. (2011). Somatic mutations of *PPP2R1A* in ovarian and uterine carcinomas. Am. J. Pathol..

[39] Calin G.A., di Iasio M.G., Caprini E., Vorechovsky I., Natali P.G., Sozzi G., Croce C.M., Barbanti-Brodano G., Russo G., Negrini M. (2000). Low frequency of alterations of the α (PPP2R1A) and β (PPP2R1B) isoforms of the subunit A of the serine-threonine phosphatase 2A in human neoplasms. Oncogene.

[40] Ruediger R., Ruiz J., Walter G. (2011). Human cancer-associated mutations in the Aα subunit of protein phosphatase 2A increase lung cancer incidence in Aα knock-in and knockout mice. Mol. Cell. Biol..

[41] Baysal B.E., Farr J.E., Goss J.R., Devlin B., Richard C.W. (1998). Genomic organization and precise physical location of protein phosphatase 2A regulatory subunit A beta isoform gene on chromosome band 11q23. Gene.

[42] Wang S.S., Esplin E.D., Li J.L., Huang L., Gazdar A., Minna J., Evans G.A. (1998). Alterations of the PPP2R1B gene in human lung and colon cancer. Science.

[43] Takagi Y., Futamura M., Yamaguchi K., Aoki S., Takahashi T., Saji S. (2000). Alterations of the PPP2R1B gene located at 11q23 in human colorectal cancers. Gut.

[44] Chou H.-C., Chen C.H., Lee H.S., Lee C.Z., Huang G.T., Yang P.M., Lee P.H., Sheu J.C. (2007). Alterations of tumour suppressor gene *PPP2R1B* in hepatocellular carcinoma. Cancer Lett..

[45] Curtis C., Shah S.P., Chin S.F., Turashvili G., Rueda O.M., Dunning M.J., Speed D., Lynch A.G., Samarajiwa S., Yuan Y. (2012). The genomic and transcriptomic architecture of 2000 breast tumours reveals novel subgroups. Nature.

[46] Cheng Y., Liu W., Kim S.T., Sun J., Lu L., Sun J., Zheng S.L., Isaacs W.B., Xu J. (2011). Evaluation of *PPP2R2A* as a prostate cancer susceptibility gene: A comprehensive germline and somatic study. Cancer Genet..

[47] The Cancer Genome Atlas Reaserch Network (2011). Integrated genomic analyses of ovarian carcinoma. Nature.

[48] Ruvolo P.P., Qui Y.H., Coombes K.R., Zhang N., Ruvolo V.R., Borthakur G., Konopleva M., Andreeff M., Kornblau S.M. (2011). Low expression of PP2A regulatory subunit B55α is associated with T308 phosphorylation of AKT and shorter complete remission duration in acute myeloid leukemia patients. Leukemia.

[49] Tan J., Lee P.L., Li Z., Jiang X., Lim Y.C., Hooi S.C., Yu Q. (2010). B55β-associated PP2A complex controls PDK1-directed myc signaling and modulates rapamycin sensitivity in colorectal cancer. Cancer Cell.

[50] Muggerud A.A., Rønneberg J.A., Wärnberg F., Botling J., Busato F., Jovanovic J., Solvang H., Bukholm I., Børresen-Dale A.L., Kristensen V.N. (2009). Frequent aberrant DNA methylation of ABCB1, FOXC1, PPP2R2B and PTEN in ductal carcinoma in situ and early invasive breast cancer. Breast Cancer Res..

[51] Bialojan C., Takai A. (1988). Inhibitory effect of a marine-sponge toxin, okadaic acid, on protein phosphatases. Specificity and kinetics. Biochem. J..

[52] Marc M. (2007). PP2A: Unveiling a reluctant tumor suppressor. Cell.

[53] Guergnon J., Godet A.N., Galioot A., Falanga P.B., Colle J.H., Cayla X., Garcia A. (2011). PP2A targeting by viral proteins: A widespread biological strategy from DNA/RNA tumor viruses to HIV-1. Biochim. Biophys. Acta.

[54] Zhao R.Y., Elder R.T. (2005). Viral infections and cell cycle G2/M regulation. Cell Res..

[55] Walter G., Ruediger R., Slaughter C., Mumby M. (1990). Association of protein phosphatase 2A with polyoma virus medium tumor antigen. Proc. Natl. Acad. Sci. USA.

[56] Pallas D.C., Shahrik L.K., Martin B.L., Jaspers S., Miller T.B., Brautigan D.L., Roberts T.M. (1990). Polyoma small and middle T antigens and SV40 small t antigen form stable complexes with protein phosphatase 2A. Cell.

[57] Campbell K.S., Auger K.R., Hemmings B.A., Roberts T.M., Pallas D.C. (1995). Identification of regions in polyomavirus middle T and small T antigens important for association with protein phosphatase 2A. J. Virol..

[58] Cristobal I., Manso R., Rincón R., Caramés C., Senin C., Borrero A., Martínez-Useros J., Rodriguez M., Zazo S., Aguilera O. (2014). PP2A inhibition is a common event in colorectal cancer and its restoration using FTY720 shows promising therapeutic potential. Mol. Cancer Ther..

[59] Letourneux C., Rocher G., Porteu F. (2006). B56-containing PP2A dephosphorylate ERK and their activity is controlled by the early gene IEX-1 and ERK. EMBO J..

[60] Silverstein A.M., Barrow C.A., Davis A.J., Mumby M.C. (2002). Actions of PP2A on the MAP kinase pathway and apoptosis are mediated by distinct regulatory subunits. Proc. Natl. Acad. Sci. USA.

[61] Rodriguez-Viciana P., Collins C., Fried M. (2006). Polyoma and SV40 proteins differentially regulate PP2A to activate distinct cellular signaling pathways involved in growth control. Proc. Natl. Acad. Sci..

[62] Hahn W.C., Dessain S.K., Brooks M.W., King J.E., Elenbaas B., Sabatini D.M., de Caprio J.A., Weinberg R.A. (2002). Enumeration of the simian virus 40 early region elements necessary for human cell transformation. Mol. Cell. Biol..

[63] Yu J., Boyapati A., Rundell K. (2001). Critical role for SV40 small-T antigen in human cell transformation. Virology.

[64] Marc M. (1995). Regulation by tumour antigens defines a role for PP2A in signal transduction. Semin. Cancer Biol..

[65] Chen W., Possemato R., Campbell K.T., Plattner C.A., Pallas D.C., Hahn W.C. (2004). Identification of specific PP2A complexes involved in human cell transformation. Cancer Cell.

[66] Sontag E., Fedorov S., Kamibayashi C., Robbins D., Cobb M., Mumby M. (1993). The interaction of SV40 small tumor antigen with protein phosphatase 2A stimulates the map kinase pathway and induces cell proliferation. Cell.

[67] Ugi S., Imamura T., Ricketts W., Olefsky J.M. (2002). Protein phosphatase 2A forms a molecular complex with Shc and regulates Shc tyrosine phosphorylation and downstream mitogenic signaling. Mol. Cell. Biol..

[68] Andrabi S., Gjoerup O.V., Kean J.A., Roberts T.M., Schaffhausen B. (2007). Protein phosphatase 2A regulates life and death decisions via Akt in a context-dependent manner. Proc. Natl. Acad. Sci. USA.

[69] Eichhorn P.J., Creyghton M.P., Wilhelmsen K., van Dam H., Bernards R. (2007). A RNA interference screen identifies the protein phosphatase 2A subunit PR55γ as a stress-sensitive inhibitor of c-SRC. PLoS Genet..

[70] Stokoe D., McCormick F. (1997). Activation of c-Raf-1 by ras and SRC through different mechanisms: Activation *in vivo* and *in vitro*. EMBO J..

[71] Kuo Y.-C., Huang K.Y., Yang C.H., Yang Y.S., Lee W.Y., Chiang C.W. (2008). Regulation of phosphorylation of Thr-308 of Akt, cell proliferation, and survival by the B55α regulatory subunit targeting of the protein phosphatase 2A holoenzyme to Akt. J. Biol. Chem..

[72] Kiely P.A., O’Gorman D., Luong K., Ron D., O’Connor R. (2006). Insulin-like growth factor I controls a mutually exclusive association of RACK1 with protein phosphatase 2A and β1 integrin to promote cell migration. Mol. Cell. Biol..

[73] Suganuma M., Fujiki H., Suguri H., Yoshizawa S., Hirota M., Nakayasu M., Ojika M., Wakamatsu K., Yamada K., Sugimura T. (1988). Okadaic acid: An additional non-phorbol-12-tetradecanoate-13-acetate-type tumor promoter. Proc. Natl. Acad. Sci. USA.

[74] Fujiki H., Suganuma M. (1993). Tumor promotion by inhibitors of protein phosphatases 1 and 2A: The okadaic acid class of compounds. Adv. Cancer Res..

[75] Lin Y.-C., Chen K.C., Chen C.C., Cheng A.L., Chen K.F. (2012). CIP2A-mediated Akt activation plays a role in bortezomib-induced apoptosis in head and neck squamous cell carcinoma cells. Oral Oncol..

[76] Junttila M.R., Puustinen P., Niemelä M., Ahola R., Arnold H., Böttzauw T., Ala-aho R., Nielsen C., Ivaska J., Taya Y. (2007). CIP2A inhibits PP2A in human malignancies. Cell.

[77] Khanna A., Kauko O., Böckelman C., Laine A., Schreck I., Partanen J.I., Szwajda A., Bormann S., Bilgen T., Helenius M. (2013). Chk1 targeting reactivates PP2A tumor suppressor activity in cancer cells. Cancer Res..

[78] Cristóbal I., Garcia-Orti L., Cirauqui C., Cortes-Lavaud X., García-Sánchez M.A., Calasanz M.J., Odero M.D. (2012). Overexpression of SET is a recurrent event associated with poor outcome and contributes to protein phosphatase 2A inhibition in acute myeloid leukemia. Haematologica.

[79] Kawada M., Amemiya M., Ishizuka M., Takeuchi T. (1999). Cytostatin, an inhibitor of cell adhesion to extracellular matrix, selectively inhibits protein phosphatase 2A. Biochim. Biophys. Acta.

[80] Wada S.I., Usami I., Umezawa Y., Inoue H., Ohba S., Someno T., Kawada M., Ikeda D. (2010). Rubratoxin A specifically and potently inhibits protein phosphatase 2A and suppresses cancer metastasis. Cancer Sci..

[81] Ugi S., Imamura T., Maegawa H., Egawa K., Yoshizaki T., Shi K., Obata T., Ebina Y., Kashiwagi A., Olefsky J.M. (2004). Protein phosphatase 2A negatively regulates insulin’s metabolic signaling pathway by inhibiting Akt (protein kinase B) activity in 3T3-L1 adipocytes. Mol. Cell. Biol..

[82] Kiely P.A., Baillie G.S., Lynch M.J., Houslay M.D., O’Connor R. (2008). Tyrosine 302 in RACK1 is essential for insulin-like growth factor-I-mediated competitive binding of PP2A and β1 integrin and for tumor cell proliferation and migration. J. Biol. Chem..

[83] Yoshizaki T., Maegawa H., Egawa K., Ugi S., Nishio Y., Imamura T., Kobayashi T., Tamura S., Olefsky J.M., Kashiwagi A. (2004). Protein phosphatase-2Cα as a positive regulator of insulin sensitivity through direct activation of phosphatidylinositol 3-kinase in 3T3-L1 adipocytes. J. Biol. Chem..

[84] Khanna A., Okkeri J., Bilgen T., Tiirikka T., Vihinen M., Visakorpi T., Westermarck J. (2011). ETS1 mediates MEK1/2-dependent overexpression of cancerous inhibitor of protein phosphatase 2A (CIP2A) in human cancer cells. PLoS ONE.

[85] Padmanabhan S., Mukhopadhyay A., Narasimhan S.D., Tesz G., Czech M.P., Tissenbaum H.A. (2009). A PP2A regulatory subunit regulates *C. elegans* insulin/IGF-1 signaling by modulating AKT-1 phosphorylation. Cell.

[86] Yang Y., Huang Q., Lu Y., Li X., Huang S. (2012). Reactivating PP2A by FTY720 as a novel therapy for AML with C-KIT tyrosine kinase domain mutation. J. Cell. Biochem..

[87] Cristobal I., Garcia-Orti L., Cirauqui C., Alonso M.M., Calasanz M.J., Odero M.D. (2011). PP2A impaired activity is a common event in acute myeloid leukemia and its activation by forskolin has a potent anti-leukemic effect. Leukemia.

[88] Dent P. (2013). Ceramide in the prostate. Cancer Biol. Ther..

[89] Kappos L., Radue E.W., O’Connor P., Polman C., Hohlfeld R., Calabresi P., Selmaj K., Agoropoulou C., Leyk M., Zhang-Auberson L. (2010). A placebo-controlled trial of oral fingolimod in relapsing multiple sclerosis. N. Engl. J. Med..

[90] Khatri B., Barkhof F., Comi G., Hartung H.P., Kappos L., Montalban X., Pelletier J., Stites T., Wu S., Holdbrook F. (2011). Comparison of fingolimod with interferon beta-1A in relapsing-remitting multiple sclerosis: A randomised extension of the TRANSFORMS study. Lancet Neurol..

[91] O’Connor P., Comi G., Montalban X., Antel J., Radue E.W., de Vera A., Pohlmann H., Kappos L., FTY720 D2201 Study Group (2009). Oral fingolimod (FTY720) in multiple sclerosis two-year results of a phase II extension study. Neurology.

[92] Cohen J.A., Barkhof F., Comi G., Hartung H.P., Khatri B.O., Montalban X., Pelletier J., Capra R., Gallo P., Izquierdo G. (2010). Oral fingolimod or intramuscular interferon for relapsing multiple sclerosis. N. Engl. J. Med..

[93] Brinkmann V., Billich A., Baumruker T., Heining P., Schmouder R., Francis G., Aradhye S., Burtin P. (2010). Fingolimod (FTY720): Discovery and development of an oral drug to treat multiple sclerosis. Nat. Rev. Drug discov..

[94] Matsuoka Y., Nagahara Y., Ikekita M., Shinomiya T. (2003). A novel immunosuppressive agent FTY720 induced Akt dephosphorylation in leukemia cells. Br. J. Pharmacol..

[95] Li M.H., Hla T., Ferrer F. (2013). FTY720 inhibits tumor growth and enhances the tumor-suppressive effect of topotecan in neuroblastoma by interfering with the sphingolipid signaling pathway. Pediatr. Blood Cancer.

[96] Azuma H., Takahara S., Horie S., Muto S., Otsuki Y., Katsuoka Y. (2003). Induction of apoptosis in human bladder cancer cells *in vitro* and *in vivo* caused by FTY720 treatment. J. Urol..

[97] Ubai T., Azuma H., Kotake Y., Inamoto T., Takahara K., Ito Y., Kiyama S., Sakamoto T., Horie S., Muto S. (2007). FTY720 induced Bcl-associated and Fas-independent apoptosis in human renal cancer cells *in vitro* and significantly reduced *in vivo* tumor growth in mouse xenograft. Anticancer Res..

[98] Marvaso G., Barone A., Amodio N., Raimondi L., Agosti V., Altomare E., Scotti V., Lombardi A., Bianco R., Bianco C. (2014). Sphingosine analog fingolimod (FTY720) increases radiation sensitivity of human breast cancer cells *in vitro*. Cancer Biol. Ther..

[99] Zhang N., Dai L., Qi Y., Di W., Zhang P. (2013). Combination of FTY720 with cisplatin exhibits antagonistic effects in ovarian cancer cells: Role of autophagy. Int. J. Oncol..

[100] Saddoughi S.A., Gencer S., Peterson Y.K., Ward K.E., Mukhopadhyay A., Oaks J., Bielawski J., Szulc Z.M., Thomas R.J., Selvam S.P. (2013). Sphingosine analogue drug FTY720 targets I2PP2A/SET and mediates lung tumour suppression via activation of PP2A-RIPK1-dependent necroptosis. EMBO Mol. Med..

[101] Baldacchino S., Saliba C., Petroni V., Fenech A.G., Borg N., Grech G. (2014). Deregulation of the phosphatase, PP2A is a common event in breast cancer, predicting sensitivity to FTY720. EPMA J..

[102] Roberts K.G., Smith A.M., McDougall F., Carpenter H., Horan M., Neviani P., Powell J.A., Thomas D., Guthridge M.A., Perrotti D. (2010). Essential requirement for PP2A inhibition by the oncogenic receptor C-KIT suggests PP2A reactivation as a strategy to treat C-KIT+ cancers. Cancer Res..

[103] Van Hoof C., Goris J. (2003). Phosphatases in apoptosis: To be or not to be, PP2A is in the heart of the question. Biochim. Biophys. Acta.

[104] Li W., Xie L., Chen Z., Zhu Y., Sun Y., Miao Y., Xu Z., Han X. (2010). Cantharidin, a potent and selective PP2A inhibitor, induces an oxidative stress-independent growth inhibition of pancreatic cancer cells through G2/M cell-cycle arrest and apoptosis. Cancer Sci..

[105] Schweyer S., Bachem A., Bremmer F., Steinfelder H.J., Soruri A., Wagner W., Pottek T., Thelen P., Hopker W.W., Radzun H.J. (2007). Expression and function of protein phosphatase PP2A in malignant testicular germ cell tumours. J. Pathol..

[106] Duong F.H., Dill M.T., Matter M.S., Makowska Z., Calabrese D., Dietsche T., Ketterer S., Terracciano L., Heim M.H. (2014). Protein phosphatase 2A promotes hepatocellular carcinogenesis in the diethylnitrosamine mouse model through inhibition of p53. Carcinogenesis.

[107] Lu J., Kovach J.S., Johnson F., Chiang J., Hodes R., Lonser R., Zhuang Z. (2009). Inhibition of serine/threonine phosphatase PP2A enhances cancer chemotherapy by blocking DNA damage induced defense mechanisms. Proc. Natl. Acad. Sci. USA.

[108] Boudreau R., Conrad D.M., Hoskin D.W. (2007). Apoptosis induced by protein phosphatase 2A (PP2A) inhibition in T leukemia cells is negatively regulated by PP2A-associated p38 mitogen-activated protein kinase. Cell. Signal..

[109] Santoro M.F., Annand R.R., Robertson M.M., Peng Y.W., Brady M.J., Mankovich J.A., Hackett M.C., Ghayur T., Walter G., Wong W.W. (1998). Regulation of protein phosphatase 2A activity by caspase-3 during apoptosis. J. Biol. Chem..

[110] Li X., Scuderi A., Letsou A., Virshup D.M. (2002). B56-associated protein phosphatase 2A is required for survival and protects from apoptosis in *Drosophila melanogaster*. Mol. Cell. Biol..

[111] MacKeigan J.P., Murphy L.O., Blenis J. (2005). Sensitized RNAi screen of human kinases and phosphatases identifies new regulators of apoptosis and chemoresistance. Nat. Cell Biol..

[112] Ruvolo P., Deng X., May W. (2001). Phosphorylation of Bcl2 and regulation of apoptosis. Leukemia.

[113] Ruvolo P.P., Clark W., Mumby M., Gao F., May W.S. (2002). A functional role for the B56 α-subunit of protein phosphatase 2A in ceramide-mediated regulation of Bcl2 phosphorylation status and function. J. Biol. Chem..

[114] Simizu S., Tamura Y., Osada H. (2004). Dephosphorylation of Bcl-2 by protein phosphatase 2A results in apoptosis resistance. Cancer Sci..

[115] Brown C.J., Lain S., Verma C.S., Fersht A.R., Lane D.P. (2009). Awakening guardian angels: Drugging the p53 pathway. Nat. Rev. Cancer.

[116] Ajay A.K., Upadhyay A.K., Singh S., Vijayakumar M.V., Kumari R., Pandey V., Boppana R., Bhat M.K. (2010). Cdk5 phosphorylates non-genotoxically overexpressed p53 following inhibition of PP2A to induce cell cycle arrest/apoptosis and inhibits tumor progression. Mol. Cancer.

[117] Li D.W., Liu J.P., Schmid P.C., Schlosser R., Feng H., Liu W.B., Yan Q., Gong L., Sun S.M., Deng M. (2006). Protein serine/threonine phosphatase-1 dephosphorylates p53 at Ser-15 and Ser-37 to modulate its transcriptional and apoptotic activities. Oncogene.

[118] Crescenzi E., Raia Z., Pacifico F., Mellone S., Moscato F., Palumbo G., Leonardi A. (2013). Down-regulation of wild-type p53-induced phosphatase 1 (Wip1) plays a critical role in regulating several p53-dependent functions in premature senescent tumor cells. J. Biol. Chem..

[119] Shouse G.P., Cai X., Liu X. (2008). Serine 15 phosphorylation of p53 directs its interaction with B56γ and the tumor suppressor activity of B56γ-specific protein phosphatase 2A. Mol. Cell. Biol..

[120] Li H.H., Cai X., Shouse G.P., Piluso L.G., Liu X. (2007). A specific PP2A regulatory subunit, B56γ, mediates DNA damage-induced dephosphorylation of p53 at Thr55. EMBO J..

[121] Nobumori Y., Liu X. (2014). Characterization of tumor-derived B56γ mutations and their effect on the tumor suppressor function of B56γ-PP2A (802.21). FASEB J..

[122] Duong F.H., Filipowicz M., Tripodi M., La Monica N., Heim M.H. (2004). Hepatitis C virus inhibits interferon signaling through up-regulation of protein phosphatase 2A. Gastroenterology.

[123] Sallman D.A., Wei S., List A. (2014). PP2A: The achilles heal in MDS with 5q deletion. Front. Oncol..

[124] Wei S., Chen X., Rocha K., Epling-Burnette P.K., Djeu J.Y., Liu Q., Byrd J., Sokol L., Lawrence N., Pireddu R. (2009). A critical role for phosphatase haplodeficiency in the selective suppression of deletion 5q MDS by lenalidomide. Proc. Natl. Acad. Sci. USA.

[125] List A.F., Bennett J.M., Sekeres M.A., Skikne B., Fu T., Shammo J.M., Nimer S.D., Knight R.D., Giagounidis A., MDS-003 Study Investigators (2014). Extended survival and reduced risk of AML progression in erythroid-responsive lenalidomide-treated patients with lower-risk del (5q) MDS. Leukemia.

[126] Honkanen R.E. (1993). Cantharidin, another natural toxin that inhibits the activity of serine/threonine protein phosphatases types 1 and 2A. FEBS Lett..

[127] Riordan F.A., Foroni L., Hoffbrand A.V., Mehta A.B., Wickremasinghe R.G. (1998). Okadaic acid-induced apoptosis of HL60 leukemia cells is preceded by destabilization of Bcl-2 mRNA and downregulation of Bcl-2 protein. FEBS Lett..

[128] Bonness K., Aragon I.V., Rutland B., Ofori-Acquah S., Dean N.M., Honkanen R.E. (2006). Cantharidin-induced mitotic arrest is associated with the formation of aberrant mitotic spindles and lagging chromosomes resulting, in part, from the suppression of PP2Aα. Mol. Cancer Ther..

[129] Huang Y., Liu Q., Liu K., Yagasaki K., Zhang G. (2009). Suppression of growth of highly-metastatic human breast cancer cells by norcantharidin and its mechanisms of action. Cytotechnology.

[130] Liu S., Yu H., Kumar S.M., Martin J.S., Bing Z., Sheng W., Bosenberg M., Xu X. (2011). Norcantharidin induces melanoma cell apoptosis through activation of TR3 dependent pathway. Cancer Biol. Ther..

[131] Kok S., Hong C.Y., Kuo M.Y., Lee C.H., Lee J.J., Lou I.U., Lee M.S., Hsiao M., Lin S.K. (2003). Comparisons of norcantharidin cytotoxic effects on oral cancer cells and normal buccal keratinocytes. Oral Oncol..

[132] Fan Y.-Z., Fu J.Y., Zhao Z.M., Chen C.Q. (2007). Inhibitory effect of norcantharidin on the growth of human gallbladder carcinoma GBC-SD cells *in vitro*. Hepatobiliary Pancreat Dis. Int..

[133] To K.K., Ho Y.-P., Au-Yeung S.C. (2005). *In vitro* and *in vivo* suppression of growth of hepatocellular carcinoma cells by novel traditional Chinese medicine-platinum anti-cancer agents. Anticancer Drugs.

[134] Aird K.M., Ghanayem R.B., Peplinski S., Lyerly H.K., Devi G.R. (2010). X-linked inhibitor of apoptosis protein inhibits apoptosis in inflammatory breast cancer cells with acquired resistance to an ErbB1/2 tyrosine kinase inhibitor. Mol. Cancer Ther..

[135] Chung V.M., Mansfield A.S., Kovach J. (2014). A phase 1 study of a novel inhibitor of protein phosphatase 2A alone and with docetaxel. J. Clin. Oncol..

[136] Lu J., Zhuang Z., Song D.K., Mehta G.U., Ikejiri B., Mushlin H., Park D.M., Lonser R.R. (2010). The effect of a PP2A inhibitor on the nuclear receptor corepressor pathway in glioma: Laboratory investigation. J. Neurosurg..

[137] Zimmerman R., Peng D.J., Lanz H., Zhang Y.H., Danen-Van Oorschot A., Qu S., Backendorf C., Noteborn M. (2012). PP2A inactivation is a crucial step in triggering apoptin-induced tumor-selective cell killing. Cell Death Dis..

[138] McDermott M.S., Browne B.C., Conlon N.T., O’Brien N.A., Slamon D.J., Henry M., Meleady P., Clynes M., Dowling P., Crown J. (2014). PP2A inhibition overcomes acquired resistance to HER2 targeted therapy. Mol. Cancer.

[139] Geyer C.E., Forster J., Lindquist D., Chan S., Romieu CG., Pienkowski T., Jagiello-Gruszfeld A., Crown J., Chan A., Kaufman B. (2006). Lapatinib plus capecitabine for HER2-positive advanced breast cancer. N. Engl. J. Med..

[140] Liu L., Greger J., Shi H., Liu Y., Greshock J., Annan R., Halsey W., Sathe G.M., Martin A.M., Gilmer T.M. (2009). Novel mechanism of lapatinib resistance in HER2-positive breast tumor cells: Activation of AXL. Cancer Res..

[141] Rexer B.N., Ham A.J., Rinehart C., Hill S., Granja-Ingram Nde M., González-Angulo A.M., Mills G.B., Dave B., Chang J.C., Liebler D.C. (2011). Phosphoproteomic mass spectrometry profiling links SRC family kinases to escape from HER2 tyrosine kinase inhibition. Oncogene.

[142] Xia W., Bacus S., Hegde P., Husain I., Strum J., Liu L., Paulazzo G., Lyass L., Trusk P., Hill J. (2006). A model of acquired autoresistance to a potent ErbB2 tyrosine kinase inhibitor and a therapeutic strategy to prevent its onset in breast cancer. Proc. Natl. Acad. Sci. USA.

[143] Xia W., Bacus S., Husain I., Liu L., Zhao S., Liu Z., Moseley M.A., Thompson J.W., Chen F.L., Koch K.M. (2010). Resistance to ErbB2 tyrosine kinase inhibitors in breast cancer is mediated by calcium-dependent activation of RelA. Mol. Cancer Ther..

[144] Jegg A.-M., Ward T.M., Iorns E., Hoe N., Zhou J., Liu X., Singh S., Landgraf R., Pegram M.D. (2012). PI3K independent activation of mTORC1 as a target in lapatinib-resistant ERBB2+ breast cancer cells. Breast Cancer Res. Treat..

[145] Li W., Chen Z., Zong Y., Gong F., Zhu Y., Zhu Y., Lv J., Zhang J., Xie L., Sun Y. (2011). PP2A inhibitors induce apoptosis in pancreatic cancer cell line PANC-1 through persistent phosphorylation of IKKα and sustained activation of the NF-κB pathway. Cancer Lett..

